# 

**Published:** 1906-07-28

**Authors:** 


### BOOKS RECEIVED.

Andrew Reid and Co., Ltd., Newcastle-on-Tyne.

"History of the Newcastle Infirmary." By G. H. Hume, D.C.L., M.D.

Baillière, Tindall and Cox.

"Aneurysm of the Abdominal Aorta." By F. P. Nunneley, D.M., M.A.

Surgery Publishing Co., New York.

"Surgical Suggestions." By Drs. Bricknor and Moschcowitz.

J. Wright and Co., Bristol.

"Medical Annual Synoptical Index." 1899-1904.

### Hospital Saturday in Christchurch.

HOSPITAL SATURDAY in Christchurch, N.Z., held on May 26, proved a great success. About £263 was collected. The proceeds are in aid of building a consumptive sanatorium, the Government giving a subsidy of twenty-four shillings in the pound.

### The AUGUST C.M.B. EXAM.

**Nurses entering for the next Examination of the Central Midwives Board will find the following Books on Midwifery of the greatest assistance in acquiring a thoroughly well-grounded theoretical knowledge of the subject.**

**HOW TO BECOME A CERTIFIED MIDWIFE.**
By E. L. C. Appel, M.B., B.S., B.Sc. Lond. Crown 8vo., bound limp cloth, 1/- net; 1/1 post free.

**A COMPLETE HANDBOOK OF MIDWIFERY.**
By J. K. Watson, M.D. Edin., Author of "A Handbook for Nurses."
350 pp., profusely illustrated *with* 143 *Diagrams.* 6/- net; 6/4 post free.
This handbook has been specially designed to meet the requirements of Nurses entering for the C.M.B. Examinations, and its use as a text-book should do much to ensure success.

**A PRACTICAL HANDBOOK OF MIDWIFERY.**
By Francis W. Haultain, M.D.
New and Revised Edition. Profusely illustrated with Original Cuts, Tables, &c.
Handsomely bound in leather, 6/ net; 6/3 post free.

**SIMPLE INTRODUCTORY LESSONS ON MIDWIFERY.**
By M. Loane. Crown 8vo., illustrated cloth, 1/- net; 1/1 post free.
A most useful work to Nurses beginning the theoretical study of Midwifery. It also explains the meaning of most of the technical words connected with the subject.

**NURSING NOTES ON MIDWIFERY AND GYNÆCOLOGY.**
By Sister Ross, Rotunda Hospital, Dublin. 2/- net; 2/1 post free.
(Suitable size for apron pocket.)

**THE MIDWIVES' POCKET BOOK** (with Prefatory Chapter on the Midwives Bill).
By Honnor Morten. Small crown 8vo. cloth, 1/- post free.

**INSTRUCTIONS TO MIDWIVES.**
Compiled by the Midwives' Supervising Committee of the Corporation of Manchester.
Crown 8vo., 6d. net; 7d. post free.

**LECTURES ON MIDWIFERY.**
By E. Stanley Hoare, M.R.C.S., D.P.H. Lond.
Based upon the Syllabus of the Central Midwives Board. 3/6 net; 3/9 post free.
Complete in cloth portfolio, 4/6 net; 4/9 post free.

***Catalogue of Nursing Manuals sent post free on application.***

**The Scientific Press, Ltd.,**

**Publishers of Nursing Text-Books, Charts, and Case-Books of all Descriptions.**

**28 & 29 SOUTHAMPTON STREET, STRAND, LONDON, W.C.**

